# Reviews and Book Notices

**Published:** 1881-07

**Authors:** 


### Reviews and Book Notices.

ARTICLE X.—A MANUAL ON DISEASES OF THE EYE AND EAR; FOR THE USE OF STUDENTS AND PRACTITIONERS. By W. F. MITTENDORF, M.D., Surgeon to the New York Eye and Ear Infirmary, etc., etc. New York: G. P. Putnam's Sons, 1881.

This book, it seems to us, fulfills the author's promises, as set forth in the preface, better than any other manual of the same character of which we have knowledge.

Addressing itself to medical students and practitioners, and intended to convey to them practical advice and useful information, it is written in plain language and concise form. What we approve of most particularly is the exclusion from its pages of all questions which are still unsettled and under discussion; for their introduction into text books for the beginner bewilders his mind and often causes him to abandon the study entirely.

The ophthalmological part of the work deserves our special and unqualified approval. Nothing of importance is omitted. The symptoms of the diseases delineated in clear and graphic descriptions, the directions for their treatment betray the sound judgment of the expert, and in the prescriptions in which the pages abound, we find those remedies which are valued as useful and efficient by the majority of occulists.

We are very glad to find the author has not joined the ranks of the opposition to the use of silver nitrate in the treatment of blennorrhœal ophthalmia. Our experience, however, would not allow us to subscribe to his statement (p. 92) that "it is not necessary to use a stronger application than a five-grain solution of nitrate of silver once every day." We had strong reasons in many cases to attribute the ultimate success in our treatment to the use of much stronger solutions after that of five grains had been tried faithfully but unsuccessfully.

The colored plates introduced to facilitate the recognition of diseases will, in few instances, fail to accomplish this intention of the author. For instance, fig. 8, marked "conjunctivitis," looks by all means more like the picture of incipient iritis; for its most prominent features are an intense pericorneal redness and a slight irregularity of the pupil. In fig. 10, marked "granular conjunctivitis," the ocular conjunctiva is given the brownish tint it occasionally attains after long continued use of silver (Argyrosis.)

The otological part, which the author added for the convenience of students, is a condensed abstract of Roosa's work on the ear. We think, though, the author could have improved upon the original and enhanced the practical value of his abstract by adding a few lines on post-aural abscesses, and on the early signs of phlebitis of the lateral sinus, the diagnostic significance of which has recently been pointed out by Dr. J. O. Green. Also some information with reference to the relative merits of the ear-trumpets, audiphone, and kindred appliances would undoubtedly be highly appreciated by practitioners, for upon this topic they are very likely to be interrogated by friends and patients.

We are quite certain this book, by its plain, common-sense teaching, will make hosts of friends among medical students, and we hope it will convince them that they may become as competent to treat many diseased ears and eyes as to treat a sore throat, if they only pay as much attention to eye and ear dispensaries as they give to surgical and medical clinics. F. C. H.

ARTICLE XI.—THE SKIN IN HEALTH AND DISEASE. By L DUNCAN BULKLEY, M.D., Attending Physician for Skin and Venereal Diseases at the New York Hospital, Out-patient Department; Late Physician to the Skin Department, Demilt Dispensary, New York. Philadelphia: Presley Blakiston, 1880.

This excellent work is divided into four chapters. Chapter I is a *résumé* of the anatomy and physiology of the skin. Chapter II is devoted to the consideration of the "Care of the Skin in Health." This chapter is a most entertaining and instructive one. In the third section Dr. Bulkley endeavors to "give a general idea of the way in which the skin becomes diseased," and "a sketch of the more prominent affections, and the means of their recognition and prevention as far as possible." The last chapter, "Diet and Hygiene in Diseases of the Skin," is filled with sound sense, brief and to the point.

This little book is one of the very best of the series of American Health Primers. B. W. G.

ARTICLE XII.—CUTANEOUS AND VENEREAL MEMORANDA. By HENRY G. PIFFARD, A.M., M.D., Prof. Dermatology, University of the City of New York, Surgeon to Charity Hospital, etc.; and GEORGE HENRY FOX, A.M., M.D., Surgeon to the New York Dispensary; Lecturer on Diseases of the Skin, College of Physicians and Surgeons, New York, etc. Second Edition New York: Wm. Wood & Co., 1880.

Comparatively few medical men ever become familiar with the *nomenclature* of skin diseases. Only a small per centage of physicians are thoroughly informed upon this important branch of medicine. A considerable knowledge of the Greek and Latin languages, and especially the former, is *essential* to a *thorough* understanding of the vocabulary of diseases falling under the comprehensive term, DERMATOLOGY. No man, who is not fairly proficient in these languages, enjoys being brought face to face with such a word as "Trichophytasis," and to a reader whose knowledge of language is limited to the Anglo-Saxon, the idea of encountering such a word as "Telangiectasis," is anything but enjoyable.

A careful perusal of these "Memoranda" will serve to familiarize the student and practitioner with the names, as well as the symptoms and treatment of the principal diseases of the skin.

However, for a *complete* treatise upon this subject, one must consult other and more exhaustive works. B. W. G.

